# A New Algal Friendly Extract from *Euglena cantabrica* with Potential Applications in Biomedical Field

**DOI:** 10.3390/md23120453

**Published:** 2025-11-26

**Authors:** Silvia Buonvino, Carolina Trinca, Stefan Leu, Silvia Licoccia, Sonia Melino

**Affiliations:** 1 Departmental Faculty of Medicine, UniCamillus-Saint Camillus International University of Health and Medical Sciences, Via di Sant’Alessandro 8, 00131 Rome, Italy; 2 Department of Experimental Medicine, University of Rome Tor Vergata, Via Montpellier, 00133 Rome, Italy; 3 Microalgal Biotechnology Laboratory, French Associates Institute for Agriculture and Biotechnology of Drylands, The Jacob Blaustein Institutes for Desert Research, Ben-Gurion University of the Negev, Sede-Boqer Campus, Beersheba 84990, Israel; stefanleu3@gmail.com; 4 Department of Chemical Science and Technologies, University of Rome “Tor Vergata”, Via Della Ricerca Scientifica, 00133 Rome, Italy; licoccia@uniroma2.it; 5 NAST Center, University of Rome Tor Vergata, 00133 Rome, Italy

**Keywords:** *E. cantabrica*, polyphenols, frustules, breast cancer cells, osteo-differentiation, mesenchymal stem cells

## Abstract

Microalgae, such as *Euglena cantabrica*, are rich in secondary metabolites, including polyphenols, which are valued for their antioxidant and therapeutic properties. Here a rapid, cost-effective and efficient protocol using a trichloroacetic acid (TCA) solution was developed for the production of an extract from *E. cantabrica* (EuPoly). The potential environmental and biomedical applications of this new extract were evaluated. The effects of EuPoly extract were tested on normal human dermal fibroblasts (NHDFs) and on breast cancer cells of the triple-negative MDA-MB-231 cell line. EuPoly was able to increase the NHDFs survival in oxidative -stress conditions and, on the contrary, to induce a decrease in cell viability of the breast cancer cells. EuPoly was also used to functionalize frustules (FEuPoly), mesoporous silica structures from diatoms. FEuPoly were investigated for the complexation of Cu^2+^ and Ni^2+^, as new potential tools for metal-ion decontamination. Finally, the scaffolding properties of FEuPoly were here assessed in the bone marrow mesenchymal stem cells (BM-MSCs) growth and their osteo-differentiation. This study provides new insights into the sustainable valorization of algae extracts, showing that TCA *E. cantabrica* extract and functionalized frustules may serve as multifunctional, eco-friendly resources for biomedical applications, as antioxidants and cancer cell inhibitor, metal ions-trapping and tissue osteo-repair.

## 1. Introduction

The current political priorities of the European Union (EU) are focused on promoting a sustainable economic model that balances economic growth with the preservation of natural resources. The EU’s action plan supports the development and strengthening of bio-based sectors, highlighting the importance of advancing sustainable food systems and production processes, with the goal of transitioning to a circular economy [1]. Algae and microalgae, as crucial components of the marine environment, could play a significant role in addressing the EU’s priorities of overcoming the current linear and open-loop fossil fuel-based economic model. In this context, the use of algae in an increasing variety of commercial applications makes them particularly relevant to the aims of a circular bioeconomy [1], as they are capable of producing a wide range of bioactive compounds, from antioxidants and anti-inflammatory agents to antimicrobial and anticancer molecules. This ability has spurred their use in a variety of industrial applications, including pharmaceuticals, nutraceuticals, and biofuels [2,3,4,5]. Within this broad framework of high-value products, secondary metabolites derived from microalgae have garnered significant interest due to their roles in adapting to environmental stress and defending against external threats, presenting possibilities for the use of microalgae in biosensing devices [6,7]. Among microalgae, one particularly intriguing group is the euglenoids, a class of unicellular protists known for their metabolic versatility and adaptability to various environmental conditions. Euglenoids have been investigated for their potential in environmental bioremediation and bio-medical tools for the high presence of polyphenols. Polyphenols are well known for their antioxidant properties and for their chelating abilities, which are mainly thanks to their chemical structure, particularly the presence of hydroxyl groups. Among euglenoids, *E. cantabrica* has recently emerged due to its remarkable ability to shift between photosynthetic and heterotrophic modes of nutrition, allowing it to survive and thrive in diverse environments [8]. This metabolic flexibility and its resilience to environmental stressors such as fluctuating light, temperature, and nutrient availability, combined with its high intrinsic polyphenol content, make *E. cantabrica* a promising candidate for both biomedical and environmental applications [8]. Among the polyphenols identified in *E. cantabrica*, gallic acid (5.87 μg/g), protocatechuic acid (2.97 μg/g), and epicatechin have been shown to contribute significantly to its antioxidant capacity [9,10,11]. Moreover, a notable amount of caffeic acid, p-coumaric acid, and of the flavanone naringenin have been detected [12]. Polyphenols are potent scavengers of reactive oxygen species (ROS), mitigating oxidative stress, which is a major contributing factor in the pathogenesis of various chronic diseases, including neurodegenerative diseases, cardiovascular conditions, and cancer [13,14,15,16]. In recent years, various approaches for the extraction of polyphenols from algae, plants and other agri-food products have been explored [17,18]. Polyphenol-rich extracts from euglenoids have been reported to exhibit significant antioxidant activity, along with a broad range of bioactive effects, including antiviral, anti-inflammatory, anti-cancer, and wound-healing properties [19,20,21,22]. Live *Euglena gracilis* cells and their aqueous extracts were found to facilitate wound healing by enhancing re-epithelization and reducing fibroplasia, without stimulating an excessive inflammatory response [23]. Recently, it has been demonstrated that *E. gracilis*-derived extracellular vesicles enhance skin-regenerative wound healing, promoting the proliferation and migration of skin cells, thereby increasing both collagen synthesis and the expression of proliferation-associated proteins [24]. Anti-inflammatory studies on *Euglena tuba* methanolic extracts have also been performed, employing macrophage cell lines in lipopolysaccharide-induced inflammation mice models, where the extracts reduced free radical generation [25]. In recent years, interest has grown in the use of algae- and plant-derived polyphenols for cancer treatment [26,27], as these compounds, either alone or in combination, target distinct molecular pathways and exhibit enhanced therapeutic efficacy [28,29,30]. A methanolic extract of E. tuba was found to inhibit the growth of human lung (A549) and breast cancer (MCF-7) cells in vitro through ROS-mediated regulation of MAPKs (Mitogen-activated protein kinases) [31]. Finally, it was recently reported that aqueous *E. gracilis* extract can protect against tobacco smoke carcinogen-induced lung cancer by altering the gut microbiota metabolome [32].

Herein, we aimed to obtain and characterize a new polyphenol-rich extract from *E. cantabrica* and evaluate its biological activity. In particular, the extract was investigated for its potential to protect normal human dermal fibroblasts (NHDFs) from oxidative stress induced by oxygen radicals. Furthermore, the ability of the extract to affect the viability of MDA-MB-231 triple-negative breast cancer cells was also investigated.

Beyond their therapeutic properties, algal-derived polyphenols have also been investigated for their ability to bind metal ions. Indeed, the hydroxy groups in polyphenols can form strong bonds with metal ions; therefore, polyphenols could potentially be employed in the treatment of pathological conditions, such as metal overload, or heavy metal contamination, including arsenic, cadmium, chromium, lead, mercury, nickel, and copper. Heavy metal contamination in wastewater is identified as a risk to public health and the environment, and has been since the late 19th century [33], as repeated exposure to these metals leads to bioaccumulation in tissues and organs [34], consequently causing adverse effects on human health [35]. *E. gracilis* has demonstrated significant tolerance to a wide range of heavy metals, making it a promising candidate for bioremediation in environments contaminated by multiple metals [22,36,37,38]. Nickel and copper are widely distributed pollutants with both industrial and consumer-related sources. In particular, nickel is one of the most prevalent contact allergens and skin exposure to nickel ions leads to allergic dermatitis and eczema. Moreover, Ni-containing compounds have also been linked to carcinogenic effects depending on the dose, duration, and route of exposure, and are also frequently detected in ground-water, raising concerns for both environmental and human health [39]. Regarding copper ions, although they are essential in trace amounts in cells, they can become toxic when accumulated in excess. Chronic copper ion overload is implicated in several pathological conditions, most notably in Wilson’s disease, a rare genetic disorder characterized by impaired copper metabolism and accumulation in the liver, brain, and other organs, leading to hepatic and neurological symptoms [40]. In addition, elevated copper levels have been associated with oxidative stress and cellular damage, and are potentially involved in neurodegenerative diseases such as Alzheimer’s [40].

In this context, we sought to functionalize frustules of diatoms, natural three-dimensional silica structures with micro- and nano-meter-sized pores, with the algal extract, and to evaluate its ability to scavenge biologically relevant divalent met-al ions (Cu^2+^ and Ni^2+^). These ions were specifically selected due to their involvement in biological systems: Cu^2+^ can act as a pro-oxidant agent, with elevated levels contributing to oxidative stress, neurodegeneration, and inflammatory responses, while Ni^2+^ is recognized for its cytotoxic and genotoxic effects, with involvement in allergic contact dermatitis, lung fibrosis, and carcinogenesis. Given these effects, the ability to bind and remove Cu^2+^ and Ni^2+^ is of particular relevance for applications in health protection and biomedical contexts, and could enable the development of new tools to mitigate metal-induced toxicity and enhance therapeutic strategies. Moreover, we aimed to investigate the potential applications of these functionalized structures in tissue repair. In a recent study, *E. gracilis* cells and their aqueous extracts mixed with chitosan–hyaluronic acid were used to create new bioactive hydrogels, and the efficacy of these mixtures in accelerating the wound healing process was demonstrated using a mouse model with deep skin burns [23]. However, the use of euglenoids and/or their derived bioactive compounds in combination with biomaterials for tissue regeneration represents an unexplored area of research. In this context, the ability of the *E. cantabrica* extract–functionalized frustules to act as a scaffold for human mesenchymal stem cells was assessed, paving the way for investigating these new systems as natural bioactive scaffolds with potential applications in stem cell-based tissue regeneration.

In this study, a new algal friendly extract has been characterized and investigated for its effects on the cancer and stem cells and to functionalize natural silica mesoporous structures for metal-ion trapping and improving their scaffolding properties.

## 2. Results and Discussion

### 2.1. Production and Characterization of the E. cantabrica Extract

Herein, a new protocol was developed for the production of a non-protein extract from *E. cantabrica* (EuPoly) using 50% trichloroacetic acid solution (TCA). The TCA extraction process was also compared with those using other classical solvents for polyphenol extraction (see Supplementary Information, Figure S1A) with the same incubation times and temperatures. Only the extract obtained through treating *E. cantabrica* with TCA showed a green color, demonstrating better pigment extraction. The extracts were then analyzed using the Folin–Ciocalteu assay (see Supplementary Information, Figure S1B), demonstrating the TCA solution’s major ability to extract polyphenols compared to that of the other solvents used under the same experimental conditions.

**Figure 1:**
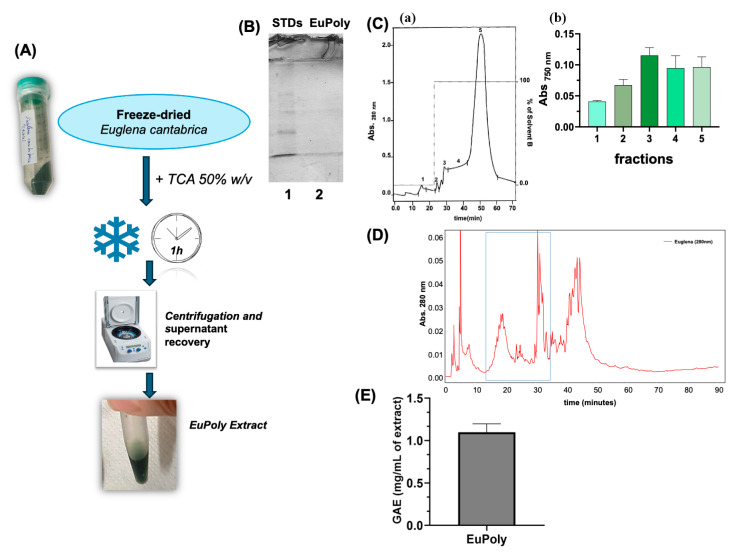
Production and Characterization of the Extract from *E. cantabrica*. (**A**) Schematic representation of *E. cantabrica* extract (EuPoly) production; (**B**) SDS-PAGE of the EuPoly extract (8 µL) (Lane 2) and molecular weight markers (Lane 1) using a 12% (*w*/*v*) polyacrylamide gel; (**C**) (a) SPE chromatogram of 40 µL of EuPoly obtained using an octadecyl column and H_2_O_dd_ as solvent A (0–22 min) and methanol as solvent B (22–70 min), the elution was recorded at 280 nm; (b) absorbance at 750 nm after Folin–Ciocalteu assay of the collected peaks (30 µL) after freeze-drying and resuspension in 100 µL of buffer; (**D**) RP-HPLC chromatogram of EuPoly obtained with the following solvent B gradient: 0–5 min 0%; 5–65 min 60%; 65–90 min 90%. The absorbance at 280 nm was monitored; (**E**) the total polyphenolic content in the extract equal to 1.097 mg/mL (S.D. ± 0.058) of polyphenols expressed as Gallic Acid Equivalents (GAE) (mg/mL of extract). Error bar indicates S.D.

Figure 1A shows the scheme of the algal extract production protocol. The absence of proteins in the extract was also demonstrated by the SDS-PAGE analysis, as shown in Figure 1B. SPE chromatography of the EuPoly was performed following the elution at 280 nm, demonstrating the high presence of hydrophobic compounds, eluted by methanol (see Figure 1C(a)). The eluted peaks were analyzed by Folin–Ciocalteu assay, further demonstrating the presence of phenolic species in the EuPoly (see Figure 1C(b)). Figure 1D shows the RP-HPLC profile of EuPoly recorded at 280 nm, where the peaks in the blue box, recorded through three RP-HPLC analyses and subsequent lyophilization, were positive to Folin–Ciocalteu assay. Their retention times are consistent with those previously reported for polyphenols [41]. The quantitative determination of polyphenols in EuPoly was performed using the Folin–Ciocalteu assay. A total poly-phenolic content of 1.097 mg/mL (S.D. ± 0.058) expressed as gallic acid equivalents (GAE) (mg/mL of extract) (Figure 1E) was determined by measuring the absorbance at 750 nm and using a calibration curve obtained with solutions at different gallic acid concentrations (0–50 μg) (see Supplementary Information, Figure S2).

In this study, the protocol for obtaining EuPoly requires the use of TCA and represents a rapid, efficient, and low-cost extraction process, which we have previously used for polyphenol extraction by vegetable-waste derived bioplastic (BPLH) [42]. The use of TCA for polyphenolic compound extraction offers advantages over other extraction methods. For instance, extraction with organic solvents, such as ethanol, methanol, acetone, or isopropanol, mixed with water is commonly used to obtain polyphenolic ex-tracts from microalgae, plants, and food industry by-products [43,44], but it often requires long incubation durations and several subsequent purification steps. Additionally, water-based or microwave-assisted extraction methods may be less selective and lead to the degradation of heat-sensitive compounds [44]. Another common technique generally used with organic solvents is ultrasound-assisted extraction, which, despite being a fast and efficient method, requires the strict selection of extraction parameters depending on the sample, which involves the frequency, ultrasound power, time, temperature, and quantity and preparation of the sample, and the type, volume, and con-centration of solvent [43]. Moreover, although TCA is classified as a toxic solvent, its use at concentrations up to 50–80% (*w*/*v*) has recently been investigated for biomedical applications, including the treatment of chronic venous ulcers and dermatological procedures for photoaging, further supporting its suitability in biomedicine and in clinical settings [45,46]. The protocol presented here provides advantages over other extraction procedures since it is rapid, cost-effective, and enables easy and efficient polyphenol extraction from *E. cantabrica*, reducing the processing time and lowering the risk of thermal degradation. Moreover, the protocol’s short incubation time (1 h) could allow for on-site extraction, providing possibilities for analysis device production. Furthermore, TCA acts as an effective protein precipitant, allowing for the ex-traction of an enriched polyphenolic fraction while minimizing the protein content. Although full characterization of the polyphenols in the extract is still needed, this innovative approach could represent a smart strategy to produce polyphenol-rich, bio-active, high-value extracts from microalgae.

### 2.2. Effects of EuPoly on Human Dermal Fibroblasts (NHDFs)

Due to the role of oxidative stress in the development of various diseases, including neurodegenerative conditions, cardiovascular disorders, and, particularly, skin pathologies (i.e., photoaging, psoriasis, and dermatitis), there is a pressing need to identify new, effective natural antioxidants with therapeutic potential. Several studies have highlighted the antioxidant characteristics of various microalgae and of many micro-algae-derived metabolites such as polyphenols, polysaccharides, and carotenoids, suggesting their potential for treating skin diseases characterized by pro-oxidative and inflammatory conditions and UV light-induced damage [47,48].

**Figure 2:**
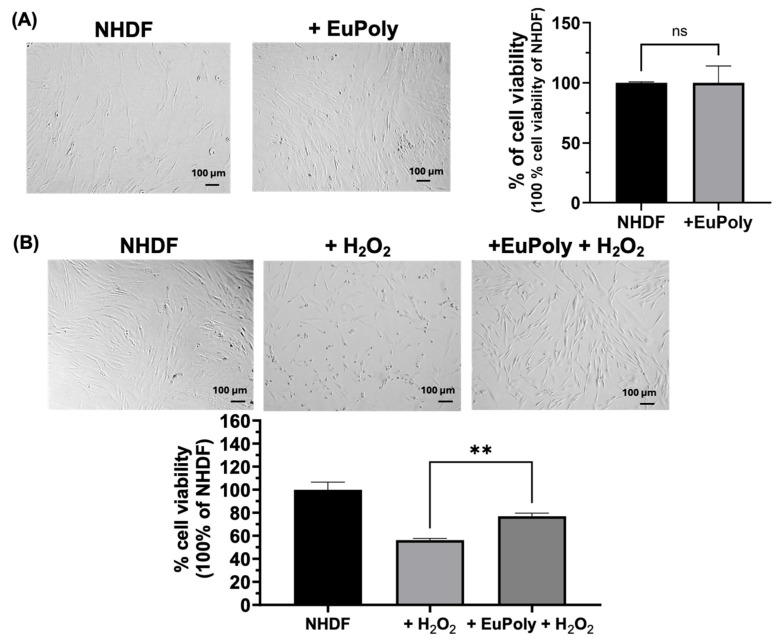
Effects of EuPoly on NHDFs’ cell viability and protection against H_2_O_2_-induced oxidative stress. (**A**) Brightfield micrographs and the cell viability assay of NHDFs treated for 24 h with EuPoly (0.5 μL/mL) added in the cell culture medium (+EuPoly) or with the same concentration of 50% TCA (NHDF); (**B**) brightfield micrographs and the cell viability assay of untreated NHDFs (NHDF), of NHDFs treated for 48 h with 100 μM of H_2_O_2_ (+H_2_O_2_) and NHDFs first treated for 3 h with EuPoly (0.5 μL/mL) and then with 100 μM of H_2_O_2_ (+EuPoly + H_2_O_2_). The results were obtained by 3 experimental replicates. Error bars indicate S.D. ns: non-significant, ** *p* value ≤ 0.01. Scale bars are of 100 μm.

The antioxidant properties of polyphenols have been extensively documented, with their mechanisms of action largely attributed to their ability to scavenge ROS, chelate metal ions, and modulate antioxidant enzyme activity [49]. Herein, the effects of EuPoly on normal human dermal fibroblasts (NHDFs) were evaluated in terms of cell viability and protection against oxidative stress. Figure 2A shows the brightfield micrographs and the results of the cell viability assay of the NHDFs after 24 h of treatment with and without EuPoly (0.5 μL/mL). No significant effects on cell viability were observed in the samples treated with EuPoly with respect to the control samples. Subsequently, the ability of EuPoly to protect NHDFs from oxidative stress induced by H_2_O_2_ treatment was investigated. NHDFs were pre-treated for 3 h with EuPoly (0.5 µL/mL), followed by treatment with 100 μM H_2_O_2_ for 48 h. As shown in Figure 2B, a 20.65 % (S.D. ± 3.44) increase in cell viability compared to cells treated with H_2_O_2_ alone was observed, demonstrating that EuPoly provides significant protection to fibroblasts against H_2_O_2_-induced oxidative stress. This effect may result from both direct ROS scavenging and an induced increase in the oxidative damage resistance of cells enabled by EuPoly.

Our results are in agreement with recent studies that have reported polyphenol-rich extracts from microalgae, including euglenoids, to significantly reduce ROS levels in vitro and suppress oxidative damage markers [50,51]. For instance, a 70% methanol *E. tuba* extract was found to have scavenging abilities against superoxide, hydroxyl, and hypochlorous acid radicals [52]. Furthermore *E. gracilis* extract has been reported to promote the cellular reducing potential and prevent lipid peroxidation in the HT-29 human intestinal epithelial cell line, promoting conditions that counteract bacterial lipopolysaccharide-induced oxidative burst [19]. Moreover, algal polyphenols and other algal molecules, such as astaxanthin and fucoidans, have been shown to activate the Nrf2/ARE signaling pathway, enhancing the expression of antioxidant enzymes like HO-1 and NQO1, thereby providing protection against oxidative stress-induced cellular damage [53,54]. Our findings show EuPoly’s antioxidant properties, highlighting its potential for further investigation as a therapeutic agent in skin disease treatment or in preventing oxidative stress-induced skin damage. Furthermore, TCA is widely used in dermatology from cosmetic peels to the treatment of actinic keratoses acne scars, warts, and at concentrations of from 40% to 60% it can stimulate the re-epithelialization process by activation of growth factors, such as TGF-α and PDGF-β [45,46]. Moreover, it is also reported to have a regulatory action on cytokines, including IL-1 and IL-10, which may also have a beneficial effect on wound healing [45]. In a dermatological study the topical application of 50% TCA on leg ulcers in 19 patients led to an evident reduction in the surface area of diabetic ulcers [45]. Therefore, EuPoly extract could combine the effect of 50% of TCA with the algal antioxidant molecules and our studies open the way for its medical application also in the treatment of diabetic ulcers.

### 2.3. Effects of EuPoly on MDA-MB-231 Breast Cancer Cells

**Figure 3:**
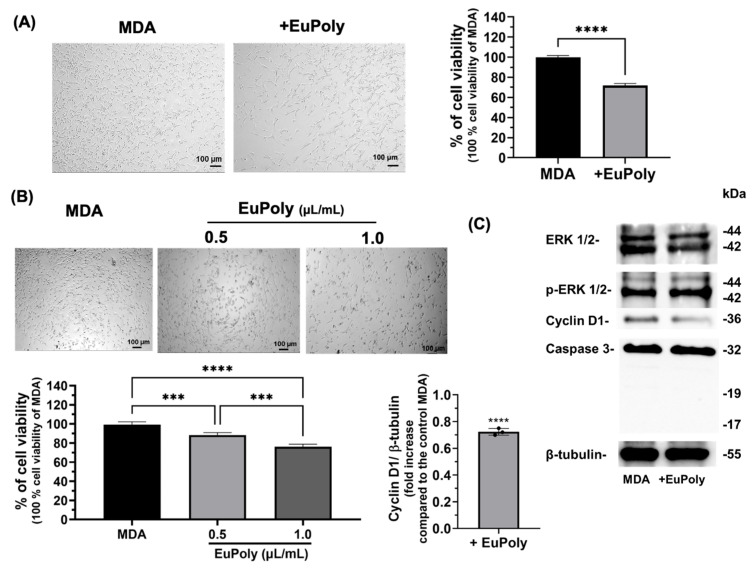
Effects of EuPoly on cell viability, proliferation and protein expression of MDA-MB-231 breast cancer cells. (**A**) Brightfield micrographs and cell counting trypan blue assay performed on MDA cells with 0.5 μL/mL of 50% TCA (MDA) and with EuPoly treatment (0.5 μL/mL) for 24 h (+EuPoly); (**B**) brightfield micrographs and WST-1 cell viability assay of MDA cells treated with 0.5 μL/mL of EuPoly (+EuPoly) added in the cell culture medium or with 0.5 μL/mL of 50% TCA (MDA) for 48 h and with 1 μL/mL of EuPoly or 50% TCA; (**C**) Western blotting analysis of Cyclin D1, p-ERK1/2, ERK1/2 and Caspase 3 protein expression of MDA cells treated with 0.5 μL/mL of EuPoly (+EuPoly) added in the cell culture medium or with 0.5 μL/mL of 50% TCA (MDA) for 48 h. The results were obtained by 3 experimental replicates. Error bars indicate S.D. *** *p* value ≤ 0.005, **** *p* value ≤ 0.0001. Scale bars are of 100 μm.

Natural polyphenols at relatively high concentrations are known to have inhibitory effects on cancer cell proliferation, tumor growth, angiogenesis, metastasis, and inflammation, and also induce programmed cell death in different types of cancer including breast, colorectal, head, lung, ovarian, melanoma, and leukemia [55]. Polyphenolic compounds, and particularly those derived from microalgae, are able to sensitize cancer cells to various therapies and, therefore, their use in combination with chemotherapy and radiotherapy has recently gathered attention [28,56]. First, the effects of EuPoly on the cell viability and proliferation of triple-negative MDA-MB-231 breast cancer cells were evaluated. Figure 3A shows the brightfield micrographs and the cell viability results as assessed using Trypan Blue after 24 h of treatment with EuPoly (0.5 μL/mL). A statistically significant decrease in cell viability of 28.21% (S.D. ± 1.56) was observed as compared to the untreated samples. Moreover, the WST-1 cell viability assay performed after 48 h of cell growth in the presence or absence of EuPoly showed that the cells treated with EuPoly at a concentration of 0.5 µL/mL exhibited a 10.82% (S.D. ± 1.86) lower viability compared to the controls, while at a concentration of 1 µL/mL, the decrease was 22.96% (S.D. ± 1.88), indicating that EuPoly was able to affect the viability of the MDA-MB-231 cancer cells in a dose-dependent manner (Figure 3B). The observed decrease in cell viability and proliferation could likely be due to cell cycle arrest induced by EuPoly, rather than programmed cell death induction, as suggested by the Western blotting analysis, which shows a reduction in Cyclin D1 expression in the EuPoly-treated samples compared to the controls and the absence of cleaved forms of Caspase 3 protein (Figure 3C). Furthermore, no significant changes in MAP kinase ERK 1/2 phosphorylation were found.

Cyclin D1 is a cell cycle protein that plays a relevant role in regulating the progression from G1 phase to the S phase of the cell cycle. Increased expression levels of this protein have been positively correlated with increased aggressiveness, invasion, and metastasis of various types of tumors, particularly triple-negative breast cancer [57,58,59,60]. Studies on different cancer cell lines have reported the role of polyphenols as growth inhibitors, either by induction of G1 cell cycle arrest, G2/M arrest, or cell death [27]. Our findings are also consistent with evidence demonstrating the ability of euglenoid extracts to exert anticancer properties. It has been observed that methanolic *E. tuba* extract is able to inhibit the in vitro growth of human lung (A549) and breast cancer (MCF-7) cells [31] and shows anticancer activity in Dalton’s lymphoma cells via reduction in mitochondrial potential and the induction of apoptosis [61]. Furthermore, an ethanol fractionated extract of *E. viridis* exerted cytotoxic activity in vitro against two prostate cancer cell lines, PC3 and Du145, and the colon cancer cell line HCT-116 [62]. Although further investigations are needed to elucidate the underlying molecular mechanisms, these findings demonstrate EuPoly’s anticancer effects on triple-negative MDA-MB-231 breast cancer cells, highlighting its potential as a safe sensitizing agent in combination with other antitumor drugs, particularly given its lack of cytotoxicity toward normal fibroblasts (Figure 2A).

### 2.4. Diatoms Frustule Functionalization with EuPoly

**Figure 4:**
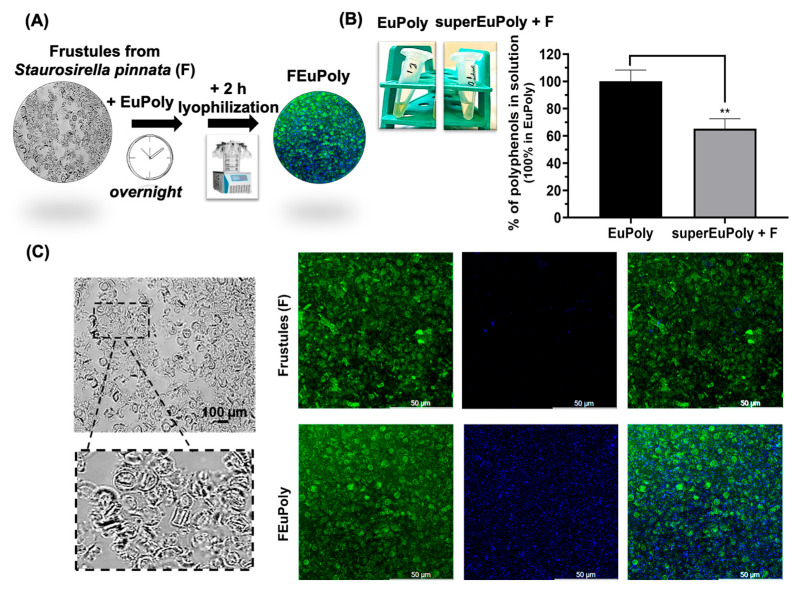
Functionalization and characterization of Frustules with EuPoly. (**A**) Schematic representation of the frustules functionalization with EuPoly to produce FEuPoly; (**B**) quantification of polyphenols by Folin–Ciocalteu assay with digital image of EuPoly and superEuPoly after incubation with frustules and centrifugation (superEuPoly + F); (**C**) brightfield micrographs of FEuPoly and confocal fluorescence micrographs of frustules with (FEuPoly) and without EuPoly functionalization (F). The green fluorescence signal (emission: 495–570 nm) was recorded using an excitation wavelength of 500 nm, while the blue fluorescence signal (emission 475–495 nm) with an excitation wavelength of 405 nm. Z-stacks were obtained with a 63× magnification and 1.5× zoom. The results were obtained by 3 experimental replicates. Error bars indicate S.D. ** *p* value ≤ 0.01. Scale bars are of 100 and 50 μm.

Diatom frustules, composed of biogenic silica, have been extensively studied for their adsorption properties and potential applications in drug delivery, biosensing, and functional material development [63,64,65]. Their unique mesoporous structure provides a high surface area and chemical versatility for functionalization. In this study, S. pinnata frustules were functionalized with the EuPoly extract, with the aim of enhancing their bioactivity and potential applications in biomedicine. After the functionalization, performed by incubating frustules overnight with EuPoly extract (FEuPoly) (Figure 4A), the samples were centrifuged and the ability of the functionalized frustules to trap polyphenols was evaluated using the Folin–Ciocalteu assay on the supernatant fraction (superEuPoly + F). A statistically significant polyphenol trapping rate of 34.84% (S.D. ± 7.38) was found (Figure 4B). FEuPoly frustules were next characterized by brightfield microscopy and confocal fluorescence microscopy (Figure 4C). Figure 4C and Video S1 show confocal micrographs of the non-functionalized frustules (F) compared to the FEuPoly frustules.

Notable autofluorescence in the green range (495–570 nm; excitation wavelength 500 nm) of the frustules was observed without and with functionalization, due to the intrinsic fluorescent property of these porous structures. The intrinsic photoluminescence of the diatom frustules in the green portion of the spectrum has been reported in the literature and specifically associated with their structural porous pattern, which resembles artificial photonic crystals and confers to frustules an active role in manipulation and exploitation of light [66,67]. However, as deduced from the confocal microscopy analysis, only the frustules functionalized with the polyphenolic extract emitted a strong fluorescence signal in the blue range of 475–495 nm when excited at 405 nm, indicating the strong presence of compounds that can be excited in the UV range and emit blue fluorescence, a characteristic typical of polyphenolic compounds that is attributed to their conjugated π-electron systems [68]. Therefore, these results represent further evidence of frustule functionalization with the EuPoly extract. The combination of frustules’ distinctive features, such as their low cost and natural availability, as well as their large specific surface area, thermal stability, biocompatibility, and customizable surface chemistry, make them highly promising for environmental applications and biomedical tools [69]. Various strategies for frustule functionalization, including metal deposition and biomolecule adsorption, have been reported [68]. In line with these approaches, our results demonstrate that the rapid functionalization of frustules using a TCA polyphenol extract is feasible, enabling the simple production of novel bioactive silica-based materials.

### 2.5. Metal Ion Trapping by FEuPoly

**Figure 5:**
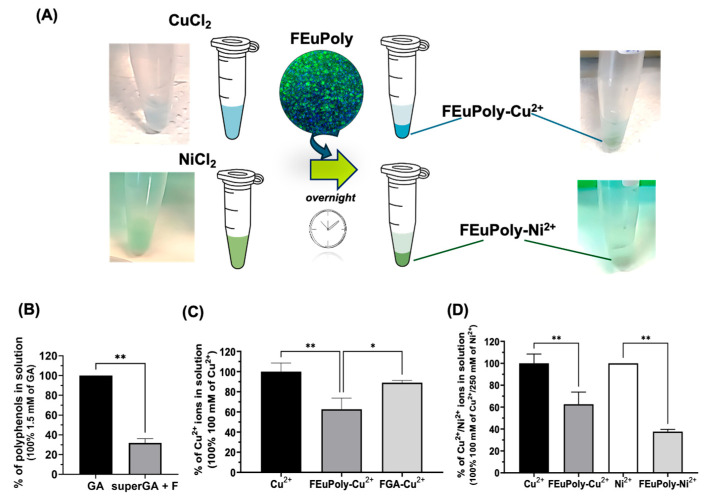
Evaluation of metal ions complexation by FEuPoly. (**A**) Schematic representation of FEuPoly’s metal-ions binding property evaluation; (**B**) quantification of polyphenols by Folin–Ciocalteu assay of GA and superGA after incubation with frustules and centrifugation (superGA + F); (**C**) quantification of Cu^2+^ ions left in solution after FEuPoly and FGA incubation with CuCl_2_ (FEuPoly-Cu^2+^ and FGA-Cu^2+^) determined measuring the absorbance at 750 nm; (**D**) quantification of Ni^2+^ ions left in solution after FEuPoly incubation with NiCl_2_ (FEuPoly-Ni^2+^) determined measuring the absorbance at 415 nm, compared to quantification of Cu^2+^ ions in solution. The results were obtained by 3 experimental replicates. Error bars indicate S.D. * *p* value ≤ 0.05, ** *p* value ≤ 0.01.

Heavy metals are among the most common pollutants; they are not biodegradable and tend to accumulate in the environment, including in water, soil, and living organisms, leading to various illnesses and health disorders [70]. Among heavy metals, both cop-per (Cu^2+^) and nickel (Ni^2+^) ions are widely used across the domestic, industrial, medicinal, and agricultural sectors [71]. Polyphenols are well known for their ability to bind metal ions, primarily due to their multiple hydroxyl (-OH) groups and catechol or galloyl structures, which can chelate metal cations [72]; for this reason, polyphenol- rich extracts from algae have recently garnered attention in the field of environ-mental remediation. Herein, we investigated the potential trapping of the divalent metal ions Cu^2+^ and Ni^2+^ by frustules functionalized with polyphenolic extract. Figure 5A shows the schematic representation of FEuPoly’s metal ion-binding property evaluation. The frustules were also functionalized with gallic acid (FGA) (Figure 5B) in order to compare its metal-binding ability with that of FEuPoly. Figure 5C shows that FEuPoly exhibited a 26.38% (S.D. ± 6.58) ± 6.59 higher Cu^2+^ binding capability com-pared to FGA, likely due to the presence of other molecules and further polyphenols with multiple functional groups (–OH, –COOH, –C=O, etc.) such as flavonoids, tannins, and floro-tannins present in the extract that are able to bind divalent metal cat-ions, enhancing the overall metal chelation rate. Interestingly, FEuPoly was found to bind Ni^2+^ more efficiently than Cu^2+,^ with a 25.03 % (S.D. ± 6.58) higher binding capability, as shown in Figure 5D. The preferential Ni^2+^ binding/coordination of FEuPoly in respect to Cu^2+^ ions could be advantageous in aquaculture or water remediation, considering the higher toxicity/allergenic properties of nickel ions than copper ions [73]. In light of the potential toxicity of these metal ions, the World Health Organization has set recommended maximum concentrations for these ions in drinking water: 31.5 µM for Cu^2+^ and 0.34 µM for Ni^2+^ [73]. Indeed, although these metals play essential roles in various biological and technological processes, excessive exposure can lead to serious health issues, including neurodegenerative diseases such as Parkinson’s and Alzheimer’s, as well as Wilson’s disease, asthma, pneumonitis, lung cancer, and disorders of the central nervous system [71].

Recently, the development of polyphenol-functionalized platforms, such as porous particles, hydrogels, capsules, and films, mainly based on synthetic polymers (i.e., PEG, PVA, and PAAM) or nanomaterials able to exploit the metal chelation properties of polyphenols, has attracted significant interest in biomedicine (i.e., drug delivery, bioimaging, materials for bio-interfacial engineering) [74,75] and environmental remediation [76,77]. In this context, the preliminary data presented here may suggest that FEuPoly represents a natural, green, and sustainable platforms potentially useful for sensing applications, or reducing the metal overload in pathological conditions associated with abnormal metal ion accumulation [78,79].

### 2.6. Evaluation of Stem Cell Scaffolding Properties of FEuPoly

**Figure 6:**
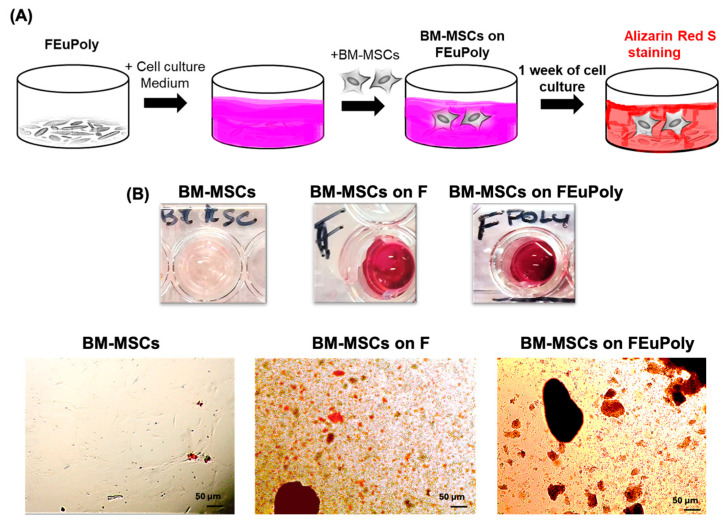
Evaluation of cell scaffolding properties of FEuPoly for cell growth and osteo-differentiation of BM-MSCs. (**A**) Schematic representation of the experimental set up to assess the osteo-differentiation of BM-MSCs grown on FEuPoly by Alizarin Red S assay; (**B**) digital images and brightfield micrographs after Alizarin Red S staining of BM-MSCs grown for 7 days on plates (BM-MSCs), on frustules (BM-MSCs on F) or on FEuPoly (BM-MSCs on FEuPoly). The results were obtained by 3 experimental replicates. Scale bars are of 50 μm.

EuPoly’s effects on the cell growth and differentiation of mesenchymal stem cells were also assessed in this study. Specifically, the potential use of EuPoly to produce new functionalized cellular scaffolds was investigated by assessing the ability of FEuPoly to support stem cell growth. Bone marrow mesenchymal stem cells (BM-MSCs) were seeded on F and FEuPoly and cultured for 7 days. Alizarin Red S staining was per-formed to detect the presence of calcium-rich deposits produced by the cells (Figure 6A). Our results revealed the strong presence of red-stained calcium deposits in the BM-MSC samples grown on F, and even greater presence was observed when the cells were grown on FEuPoly (Figure 6B). In contrast, no calcium deposits were detected in the cells grown on the plates without frustules.

The strong presence of Alizarin Red-positive deposits in the BM-MSCs grown on FEuPoly could be due to the combination of the silica frustules’ mechanical properties and the Euglena polyphenols’ biochemical activity, which creates a synergistic environment that promotes osteogenic differentiation. The use of polyphenols in tissue engineering applications has recently been investigated due to their potential ability to enhance the bioactivity and biocompatibility of scaffolds and promote cell adhesion, proliferation, and differentiation while showing anti-inflammatory, antioxidant, and antimicrobial properties, thus facilitating the tissue regeneration process. Certain polyphenols, including flavonoids, have also been reported to influence signaling path-ways relevant to wound healing and bone regeneration, suggesting a beneficial role in regenerative medicine applications [80,81,82].

Diatom silica frustules show a highly porous architecture and significant stiffness, closely resembling the structure of bone tissue. This structural mimicry provides mechanical cues that are critical for directing stem cell fate and promoting their osteo-genic differentiation by activating mechano-transduction pathways, such as the Wnt/β-catenin and TGF-β/BMP signaling cascades [83,84]. Furthermore, silica frustules’ porosity can enhance cell adhesion, infiltration, and proliferation, also addressing the limit of oxygen deprivation and facilitating bone-specific extracellular matrix component deposition. Recently, diatoms were 3D bio-printed with stem cells in a gelatin methacryloyl (GelMA) bio-ink for bone repair in animal models, and the frustules present in the diatoms were able to reduce hypoxia damage and promote bone regeneration [85]. Therefore, frustule functionalization with the algal extract proposed in our study could be a novel approach for enhancing the osteogenic–regenerative properties of these natural mesoporous structures. Moreover, polyphenols alone have also been found to contribute to osteogenic differentiation by modulating key signaling pathways involved in bone formation [86,87,88]. For instance, flavonoids have been shown to modulate the ability of MSCs to self-renew and their osteogenic differentiation potential by targeting multiple signaling pathways such as the Wnt/β-catenin pathway, ERK pathway, and PI3K/Akt pathway, and regulating bone-specific markers and transcription factors including ALP, Runx2, BMP-2, Cbfa1, and Osx [86,87,88]. In recent years, polyphenol-functionalized scaffolds including hydrogels, films, and nano-fibers, mainly based on synthetic polymers, have emerged [80]. For instance, Lee et al. developed functionalized scaffolds based on different polymers, such as polystyrene and polydimethylsiloxane, for improving osteo-regeneration abilities of human adipose-derived stem cells through surface modification via catechin coating [89]. Although these emerging synthetic polyphenol-functionalized platforms have shown promise, our FEuPoly cell scaffold, as a fully natural, microalgae-derived system, potentially offers a more sustainable and biomimetic alternative, thus representing an innovative system with promising applications in biomedicine and bone tissue regeneration.

## 3. Materials and Methods

### 3.1. Preparation of Dried Algal Biomass

*E. cantabrica* (https://utex.org/products/utex-lb-1320, accessed on 20 May 2025) was obtained from the University of Texas’s culture collection. The alga was cultured under photoautotrophic conditions in BG11 medium at 80 microeinstein light intensity in 4 cm glass bubble columns or at 200 microoeinstein light intensity in 8 cm glass bubble columns placed in a water bath at a temperature of 25 °C. The first inoculum had to be prepared at a 5-fold lower light intensity until the green mobile Euglena cells emerged into the liquid medium. Cells were grown to about 2 g/L and harvested in a Sorvall tabletop centrifuge. Pellets were rinsed with deionized water, quick-frozen and stored at −80 °C, and lyophilized using a VirTis Benchtop 2K ES Freeze Dry System (Steroglass, Perugia, Italy).

### 3.2. Production of the E. cantabrica Extract (EuPoly) and Its Characterization by SPE, RP-HPLC and SDS-PAGE Analyses

The extract (EuPoly) was obtained from freeze-dried *E. cantabrica*. Approximately 12 mg of the lyophilized algae were resuspended in 500 µL of a 50% trichloroacetic acid (TCA) (*w*/*v*) solution and incubated on ice for 1 h. Subsequently, the samples were centrifuged at 8000× *g* for 15 min at 4 °C, and the supernatants were recovered for analysis. The obtained extract was stored at −20 °C. SPE chromatography of the extract was performed using a Bakerbond SPE oc-tadecyl column (J.T.Baker, Deventer, The Netherlands) [90]; the solvents used were H_2_O_dd_ as solvent A and HPLC-grade methanol (Sigma-Aldrich, Milan, Italy) as solvent B. The chromatography was performed through isocratic elution 0–22 min at 0% of solv. B and then at 100% of solv. B (22–70 min) using a peristaltic pump, the elution was detected using an UV detector at 280 nm (Pharmacia LKB, Varese, Italy). The eluted fractions by SPE chromatography were collected, lyophilized, and then analyzed using the Folin–Ciocalteu assay. The extract was also characterized via reversed-phase high-performance liquid chromatography (RP-HPLC). RP-HPLC analysis of EuPoly was performed using mod. LC-10AVP (Shimadzu, Milan, Italy), equipped with a UV detector (Shimadzu, Milan, Italy) and a C_18_ column (150 mm × 4.6 mm, 5 μm, CPS Analitica, Rome, Italy), a loop of 20 μL, and a rate flow 0.8 mL/min. The solvents used were solvent A (0.1% (*v*/*v*) TFA) and solvent B (70% (*v*/*v*) CH_3_CN, 0.1% (*v*/*v*) TFA in H_2_O_dd_). The solvent B gradient was 0–5 min 0%; 5–65 min 60%; 65–90 min 90%. The elution was monitored at 280 nm. The eluted peaks of three RP-HPLC analyses were collected, lyophilized, and then analyzed using the Folin–Ciocalteu assay. An SDS-PAGE analysis of the EuPoly extract was performed using a 12% *w*/*v* of polyacrylamide gel, and using a sample buffer with 2 μL of 1.5 M Tris-HCl, pH 8.8, for increasing the pH of the samples.

### 3.3. Quantitative UV Spectrophotometric Analysis for Polyphenolic Content Determination

Polyphenol quantification was performed using a modified Folin–Ciocalteu assay [91]: 250 μL of Folin–Ciocalteu reagent (diluted 1:10 *v*/*v*) was added to 30 μL of TCA extract for 4 min at room temperature in dark conditions. After that, 20 μL of 1 M KOH and 200 μL of 1 M NaHCO_3_ were added and the samples were incubated in the dark for 2 h at room temperature. The absorbance at 750 nm of 200 μL of each sample was then recorded using an iMark^TM^ Microplate Absorbance Reader (Bio-Rad, Milan, Italy). A calibration curve (see Supplementary Materials Figure S2) using gallic acid (GA) was created in order to assess the total polyphenol content in GA equivalents (GAE) (mg/mL of extract).

### 3.4. Cell Cultures and WST-1 Cell Viability Assay

Cell studies were performed on MDA-MB-231 cells, NHDFs (Lonza, Basel, Switzerland), and BM-MSCs (Gibco, StemPro^®^, Life Technologies, Monza, Italy). The cell lines were cultured at 37 °C, under 5% CO_2_, in high-glucose Dulbecco’s Modified Eagle’s Medium (DMEM) (Gibco, Monza, Italy), containing 10% Fetal Bovine Serum (FBS) (*v*/*v*) (Gibco, Monza, Italy), 1% penicillin–streptomycin (*w*/*v*) (Sigma-Aldrich, Milan, Italy), and 2 mM of L-Glutamine solution (Gibco, Italy). The brightfield microscopy of the cell cultures was performed using a Zeiss microscope (Primovert, Zeiss, Milan, Italy). Cell viability was assessed by counting MDA-MB-231 breast cancer cells with Trypan Blue staining after 24 h of cell growth.

The cell viability of NHDFs and of MDA-MB-231 cells was assessed using a WST-1(4-[3-(4-lodophenyl)-2-(4-nitrophenyl)-2H-5-tetrazolium]-1,3-benzene disulfonate (Cell Proliferation Reagent WST-1, Roche, Mannheim, Germany) assay [92]. NHDFs were seeded at a density of 2 × 10^4^ cells/cm^2^. After 24 h of cell growth, the medium was changed, and cells were treated with 100 μM H_2_O_2_ for 48 h with and without a previous incubation with EuPoly 0.5 μL/mL (0.55 μg/mL of GAE) for 3 h. After, the medium was replaced with fresh high-glucose DMEM without phenol red (GIBCO, Italy) containing tetrazolium salt WST-1 (5% *v*/*v*); the cells were incubated for 2 h at 37 °C, under 5% CO_2_, and the absorbance of the medium was evaluated at 450 nm using a microplate reader (iMark^TM^ Microplate Absorbance Reader, Bio-Rad, Milan, Italy). MDA-MB-231 breast cancer cells were seeded at a cell density of 2 × 10^4^ cells/cm^2^, and the next day, the cells were treated at different EuPoly extract concentrations (0.5 and 1 μL/mL of EuPoly, corresponding to 0.55 μg/mL and 1.1 μg/mL of GAE). Cell viability was then assessed using the WST-1 assay at 48 h of cell growth, as described above. For each cell treatment with EuPoly, control samples with the same TCA concentration (50% *w*/*v*) were included in all experiments.

250 μL of Folin–Ciocalteu reagent (diluted 1:10 *v*/*v*) was added to 30 μL of TCA extract for 4 min at room temperature in dark conditions. After that, 20 μL of 1 M KOH and 200 μL of 1 M NaHCO_3_ were added and the samples were incubated in the dark for 2 h at room temperature. The absorbance at 750 nm of 200 μL of each sample was then recorded using an iMark^TM^ Microplate Absorbance Reader (Bio-Rad). A calibration curve (see Supplementary Materials Figure S2) using gallic acid (GA) was created in order to assess the total polyphenol content in GA equivalents (GAE) (mg/mL of extract).

### 3.5. Western Blotting Analysis of Protein Expression

Proteins were extracted from 2 × 10^6^ MDA-MB-231 cells per sample using RIPA buffer (50 μL) containing a protease inhibitor cocktail (Sigma-Aldrich, Milan, Italy) and per-vanadate (Sigma-Aldrich, Milan, Italy) as a phosphatase inhibitor for the cell lysis. After incubation for 90 min on ice, lysates were centrifuged for 10 min at 8000× *g* at 4 °C. The bicinchoninic acid (BCA) protein assay (Sigma-Aldrich, Milan, Italy) was used to determine the protein content, and SDS-PAGE for cell extracts (30 μg of protein) was performed using 15% polyacrylamide gel. Electroblotting was performed on PVDF membranes using a semi-dry Trans-Blot Turbo Transfer System (Bio-Rad). After blot-ting, the membrane was blocked and probed with the following primary monoclonal antibodies: Ab-cyclin D1 rabbit (2922S, Cell Signaling Technology, Danvers, MA, USA); Caspase 3 rabbit (D3R6Y, Cell Signaling Technology, USA); Ab-p-ERK1/2 rabbit (Anti-p-ERK1 (pThr202/pTyr204) and ERK2 (pThr185/pTyr187), ab136926 Abcam, Milan, Italy); and Ab-ERK1/2 rabbit (M5670, Sigma-Aldrich, Italy). Immunoblots were then incubated with the rabbit secondary antibody (dilution 1:3000) (7074S, Cell Signaling Technology, USA) for 4 h at room temperature. Immunoblots with Ab-β-tubulin–horseradish peroxidase conjugates (AB21058, Abcam, Milan, Italy) were also probed for controlling the protein loading. A SuperSignal West Pico kit (Thermo Scientific, Waltham, MA, USA) was used to visualize signals, followed by exposure to a FluorChem Imaging system (Alpha Innotech Corporation-Analitica De Mori, Milan, Italy).

### 3.6. Frustule Preparation, EuPoly Functionalization (FEuPoly), and Metal Ion Binding

*Staurosirella pinnata* biomass harvesting was carried out after 7 days of growth, and freeze-dried biomass was treated with a boiling acid mix (H_2_SO_4_:HNO_3_:H_2_O = 3:1:1), washed in bi-distilled water (H_2_O_dd_), and then freeze-dried according to a previously published protocol [93]. Frustules (2 mg) were functionalized by overnight incubation using 50 µL of EuPoly and 150 µL of H_2_O_dd_. The resulting pellet was resus-pended in 1 mL of 50 mM HEPES buffer, centrifuged at 2000 rpm for 5 min, and then lyophilized for 2 h. For the metal ion complexation analyses, samples of functionalized frustules (FEuPoly) were treated with 200 µL of either 100 mM CuCl_2_ in 10 mM HEPES buffer, pH 5.5, or 100 mM NiCl_2_, pH 5.5, and were left to oscillate in the dark overnight. The absorbances of the supernatants from the samples were measured at 750 nm and 415 nm, respectively, using the iMarkTM Microplate Reader (Bio-Rad). The brightfield microscopy analysis of frustules was performed using a BA310 Motic microscope (Motic, Hackensack, NJ, USA). Metal ion complexation was also assessed on frustules functionalized with gallic acid (FGA). Briefly, frustules were incubated overnight with 50 µL of a gallic acid (GA) solution (1 mg/mL) and 150 µL of H_2_O_dd_. After functionalization, the same protocol described for FEuPoly was used to perform Cu^2+^ ion complexation analyses.

### 3.7. Confocal Microscopy Analysis of FEuPoly

After frustule functionalization with EuPoly and centrifugation at 1342× *g* for 5 min, the pellet was washed with H_2_O_dd_ and confocal fluorescence microscopy analysis of FEuPoly was performed using the Leica Stellaris platform. The excitation wavelength was set to 500 nm with an emission range of 495–570 nm (green), and to 405 nm with emission in the 475–495 nm range (blue).

### 3.8. FEuPoly Validation for BM-MSC Growth and Alizarin Red S Staining

FEuPoly was validated as a scaffold for BM-MSCs by seeding mesenchymal stem cells at a density of 6 × 10^3^ cells/cm^2^. After 7 days of cell culture, Alizarin Red S staining was performed to detect the presence of calcium-rich deposits. Alizarin Red S staining was performed as follows: the culture medium was removed, the well was washed twice with PBS, and cells were fixed with 4% PFA (paraformaldehyde) for 20 min in the dark. Then, after washing twice with H_2_O_dd_, the Alizarin Red S staining solution, at pH 4.1 (Sigma-Aldrich, Italy), was added for 3 min. Subsequently, the staining solution was removed, the samples were washed 3 times with H_2_O_dd_, and the red deposits were analyzed by optical microscopy using a BA310 Motic microscope (Motic, Hackensack, NJ, USA).

### 3.9. Statistical Analysis

GraphPad Prism version 8.0 for Windows (GraphPad Software, San Diego, CA, USA) was used for the statistical analysis. Data obtained from three or more experimental independent biological replicates were quantified and analyzed for each variable using a t-test or one-way ANOVA test followed by Tukey’s multiple comparisons test. A *p*-value < 0.05 was considered statistically significant. Data are presented as mean ± standard deviation (S.D.).

## 4. Conclusions

Herein, we have described a new *E. cantabrica*-derived polyphenolic extract and investigated its potential use for biomedical applications. The successful development of a rapid and cost-effective extraction protocol highlights its scalability and applicability within a circular bioeconomy framework, offering advantages for analysis device production, which requires fast and efficient on-site ex-traction procedures. Furthermore, our results demonstrated that EuPoly was able to protect human fibroblasts from ROS-induced oxidative stress and to reduce the viability of MDA-MB-231 triple-negative breast cancer cells, suggesting its good potential for treating oxidative stress-related skin disorders and as an antitumor therapy. Although a full characterization of EuPoly is required and other bioactive compounds may contribute to the observed cellular effects described here, it is reasonable to attribute these effects primarily to the extract’s polyphenolic content, also consistent with the extensive literature reporting polyphenols’ antioxidant and anticancer properties. Herein, we have also functionalized diatom frustules with EuPoly, which showed good coordination of the heavy metal divalent ions Cu^2+^ and Ni^2+^, suggesting promise for wastewater remediation and biomedical applications. Moreover, FEuPoly frustules were able to support the osteogenic differentiation of BM-MSCs, potentially offering a fully natural, microalgae-derived green scaffold for use in bone tissue re-generation. These findings highlight the innovative properties of the FEuPoly system, which combines the natural and unique mesoporous structure of diatom silica with the biochemical activity of a readily obtainable Euglena-derived polyphenolic extract for driving the osteo-differentiation of mesenchymal stem cells in bone tissue repair. In conclusion, our work contributes to the recent efforts directed towards harnessing the multifunctionality of algal bioproducts with the aim of providing new eco-sustainable tools for future applications in biomedicine.

## Data Availability

The authors declare that all data generated in this study are available within the article or the Supplementary Information. Other data related to this work are available from the corresponding authors upon request.

## Supplementary Materials

The following supporting information can be downloaded at: https://www.mdpi.com/article/10.3390/md23120453/s1, Figure S1: *E. cantabrica* extracts in different solvents; Figure S2: Folin–Ciocalteu assay of EuPoly and Gallic Acid calibration curve; Video S1: 3D confocal microscopy of FEuPoly.

## Abbreviations

The following abbreviations are used in this manuscript:

ALP	Alkaline Phosphatase
BCA	Bicinchoninic acid
BM-MSCs	Bone marrow-mesenchymal stem cells
BMP	Bone morphogenetic protein
Cbfa1	Core Binding Factor 1
DMEM	Dulbecco’s Modified Eagle Medium
ERK	Extracellular Signal-Regulated Kinase
EU	European Union
EuPoly	*Euglena cantabrica* extract
F	Frustules
FBS	Fetal Bovine Serum
FEuPoly	Frustules functionalized with *Euglena cantabrica* extract
GA	Gallic acid
GAE	Gallic acid equivalents
GelMA	Gelatin methacryloyl
HO-1	Heme Oxygenase-1
MAPKs	Mitogen-activated protein kinases
MSCs	Mesenchymal stem cells
NHDFs	Normal human dermal fibroblasts
NQO1	NAD(P)H:quinone oxidoreductase 1
Nrf2/ARE	Nuclear factor erythroid 2-related factor 2/ Antioxidant Response Element
Osx	Osterix
PAAM	Polyacrylamide
PBS	Phosphate Buffered Saline
PEG	Poly(ethylene glycol)
PFA	Paraformaldehyde
PI3K/Akt	Phosphatidylinositol 3-kinase/protein kinase B
PVA	Polyvinyl alcohol
PVDF	Polyvinylidene difluoride
ROS	Reactive oxygen species
RunX2	RUNT-related transcription factor 2
SDS-PAGE	Sodium dodecyl sulfate-polyacrylamide gel electrophoresis
TCA	Trichloroacetic acid
TFA	Trifluoroacetic acid
TGF-β	Transforming growth factor-beta
Wnt	Wingless-related integration site
