# Supplementary material for: A New Algal Friendly Extract from Euglena cantabrica with Potential Applications in Biomedical Field
Source: Mar Drugs. 2025 Nov 26;23(12):453. doi: 10.3390/md23120453 (PMC12734511; doi:10.3390/md23120453)
Supplement: Supplementary file 1 [file marinedrugs-23-00453-s001.zip › marinedrugs-3997352-supplementary.pptx]

## Slide 1
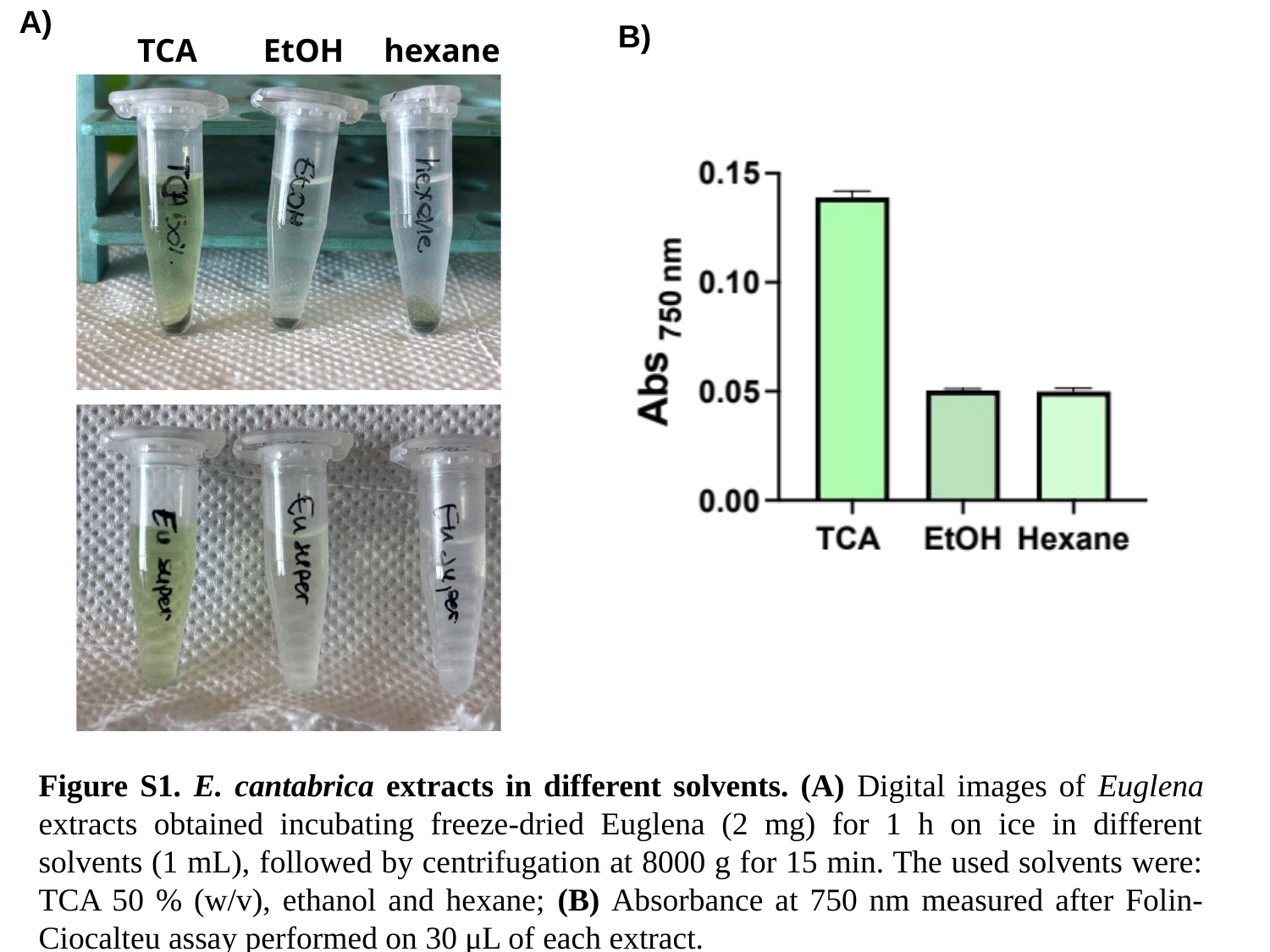

A)
B)
TCA
EtOH
hexane
Figure S1. E. cantabrica extracts in different solvents. (A) Digital images of Euglena extracts obtained incubating freeze-dried Euglena (2 mg) for 1 h on ice in different solvents (1 mL), followed by centrifugation at 8000 g for 15 min. The used solvents were: TCA 50 % (w/v), ethanol and hexane; (B) Absorbance at 750 nm measured after Folin-Ciocalteu assay performed on 30 μL of each extract.

## Slide 2
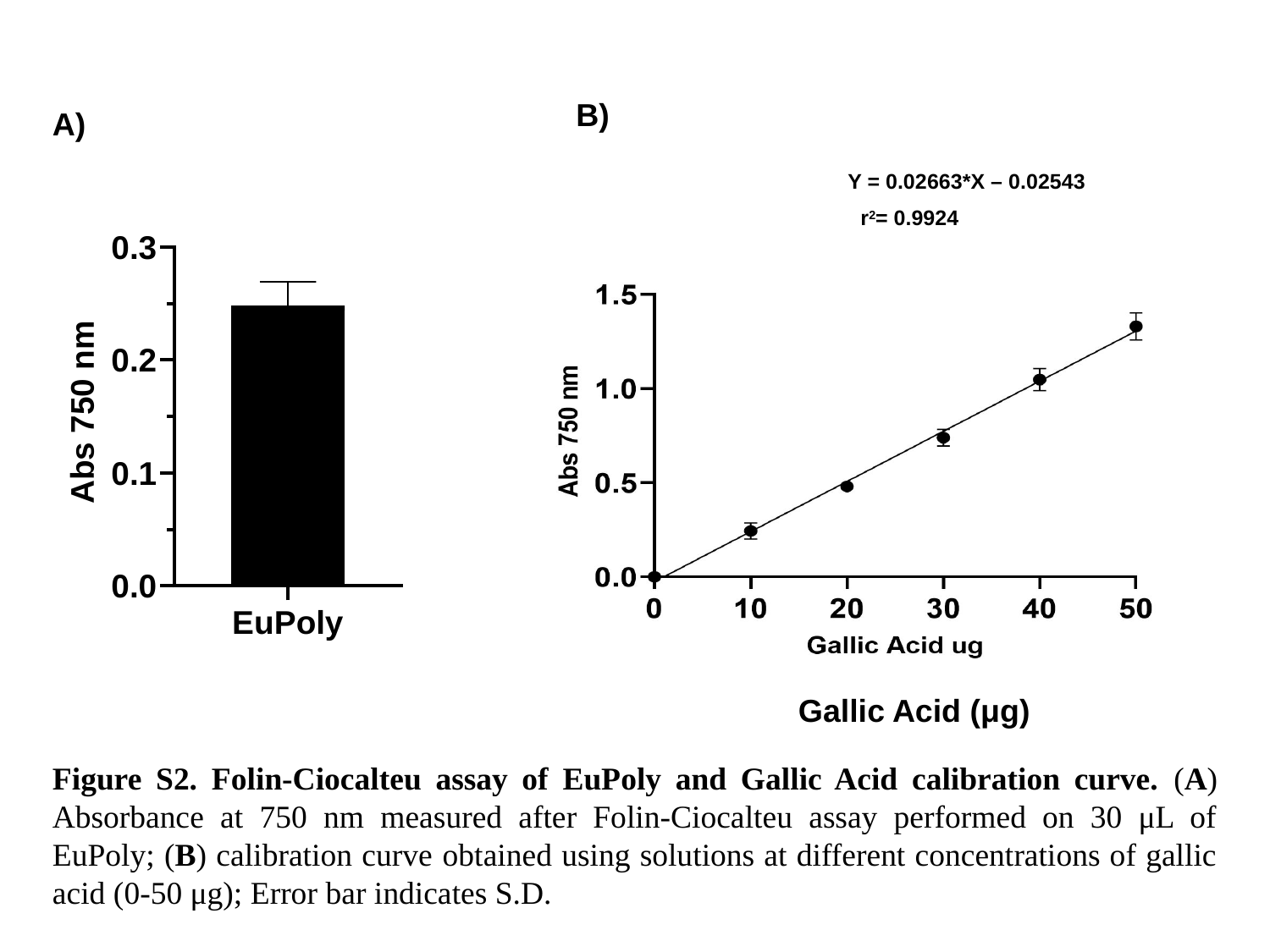

B)
A)
| Y = 0.02663\*X – 0.02543 |
| --- |
r2= 0.9924
Gallic Acid (μg)
Figure S2. Folin-Ciocalteu assay of EuPoly and Gallic Acid calibration curve. (A) Absorbance at 750 nm measured after Folin-Ciocalteu assay performed on 30 μL of EuPoly; (B) calibration curve obtained using solutions at different concentrations of gallic acid (0-50 μg); Error bar indicates S.D.

## Slide 3
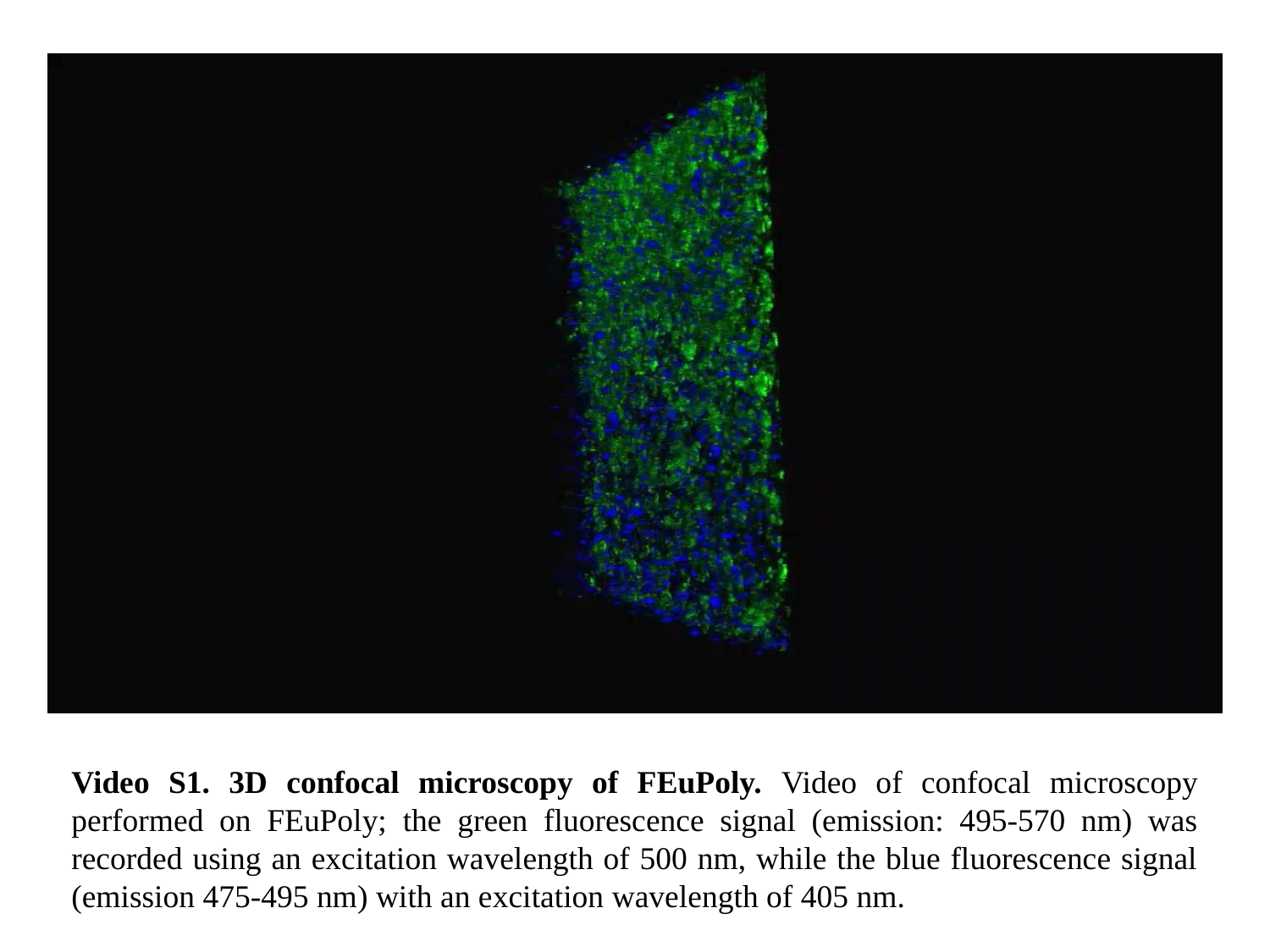

Video S1. 3D confocal microscopy of FEuPoly. Video of confocal microscopy performed on FEuPoly; the green fluorescence signal (emission: 495-570 nm) was recorded using an excitation wavelength of 500 nm, while the blue fluorescence signal (emission 475-495 nm) with an excitation wavelength of 405 nm.
